# The Power of the Web: A Systematic Review of Studies of the Influence of the Internet on Self-Harm and Suicide in Young People

**DOI:** 10.1371/journal.pone.0077555

**Published:** 2013-10-30

**Authors:** Kate Daine, Keith Hawton, Vinod Singaravelu, Anne Stewart, Sue Simkin, Paul Montgomery

**Affiliations:** 1 Centre for Evidence Based Intervention, University of Oxford, Oxford, United Kingdom; 2 Centre for Suicide Research, University of Oxford Department of Psychiatry, Warneford Hospital, Oxford, United Kingdom; 3 Oxford Health NHS Foundation Trust, Warneford Hospital, Oxford, United Kingdom; University of Granada, Spain

## Abstract

**Background:**

There is concern that the internet is playing an increasing role in self-harm and suicide. In this study we systematically review and analyse research literature to determine whether there is evidence that the internet influences the risk of self-harm or suicide in young people.

**Methods:**

An electronic literature search was conducted using the PsycINFO, MEDLINE, EMBASE, Scopus, and CINAHL databases. Articles of interest were those that included empirical data on the internet, self-harm or suicide, and young people. The articles were initially screened based on titles and abstracts, then by review of the full publications, after which those included in the review were subjected to data extraction, thematic analysis and quality rating.

**Results:**

Youth who self-harm or are suicidal often make use of the internet. It is most commonly used for constructive reasons such as seeking support and coping strategies, but may exert a negative influence, normalising self-harm and potentially discouraging disclosure or professional help-seeking. The internet has created channels of communication that can be misused to ‘cyber-bully’ peers; both cyber-bullying and general internet use have been found to correlate with increased risk of self-harm, suicidal ideation, and depression. Correlations have also been found between internet exposure and violent methods of self-harm.

**Conclusions:**

Internet use may exert both positive and negative effects on young people at risk of self-harm or suicide. Careful high quality research is needed to better understand how internet media may exert negative influences and should also focus on how the internet might be utilised to intervene with vulnerable young people.

## Introduction

The media has received considerable attention for its possible role in contributing to suicide through contagion, predominantly in youth [1], [2]. While initial attention focused on newspapers, TV and films [3], [4], [5], increasingly attention has turned to the internet [6], [7], because it is accessible, affordable in most countries, and largely unmonitored.

Despite concern about its negative impact, the potential support provided by the internet should not be overlooked [8]. Acceptance, alleviation of loneliness and shame, and creation of intimacy from internet sources may be factors that mitigate destructive desires. The internet may provide a bridge for vulnerable adolescents, giving them instant access to social networks and providing the anonymity to create or discard identities. Yet it is only speculation whether the internet is providing a safe haven, or a space where dangerous behaviours are normalised and encouraged.

Adolescence can be a time of turmoil, with high levels of stressful circumstances, developmental issues, and psychopathology, and hence of self-harm [9]. Self-harm, defined as intentional self-injury or self-poisoning, irrespective of type of motivation or extent of suicidal intent [10], usually begins and becomes most frequent between the ages of 13 and 15 years, particularly in girls [11]. Suicide is the second most common cause of death for young people worldwide and the most common cause in females aged 15–19 years [12].

Internet use increased globally by 566.4% between December 2000 and June 2012 [13]. The freedom afforded by online discussion and activity may enhance the potential for the internet to exert positive and/or negative effects on users’ psychological health [7]. Vulnerable adolescents, susceptible to bullying, victimisation and social exclusion, may be most at risk of the negative influences of the internet.

There has been much speculation and media reporting about suicide contagion and suicide pacts originating from the internet. Although there is strong evidence indicating that suicide contagion is a significant phenomenon [14], [15], direct associations with internet use are unclear. Previous research has focused on how the internet is used by suicidal people and what information is available [6], [7], [16]. Studies are, however, often rapidly outdated, and may be speculative and not systematic, or reliant on evidence from single case studies. An analysis of current evidence is needed in order to better understand the type of influence that the internet may have on young people at risk for self-harm or suicide, and to directly inform future intervention and prevention research. We have therefore conducted a systematic review of current empirical evidence on possible influences of internet use on the risk of self-harm or suicide in young people under the age of 25 years. Specifically, our aim was to identify the extent of evidence about both negative and positive influences of the internet on the risk of self-harm and suicide in young people.

## Methods

This review adheres to the guidelines detailed by the Cochrane Collaboration Handbook for Systematic Reviews [17].

An electronic literature search was conducted on December 26^th^ 2011 for all articles printed between 1991 and 2011, in the English language. 1991 was chosen as the starting date for the review as August 1991 was the date on which the internet was made available as a public service. (http://www.slac.stanford.edu/history/earlyweb/history.shtml). The databases searched were PsycINFO, MEDLINE, EMBASE, Scopus, and CINAHL. The following terms were searched for in titles, abstracts and subject headings to maximise sensitivity:

‘Internet’, ‘blog’, ‘online social network’, ‘website*’, ‘chat room*’, ‘online forum*’, ‘virtual’, ‘cyber*’, ‘web site*’, ‘facebook’, ‘twitter’, ‘myspace’, ‘bebo’, ‘online’, ‘world wide web’, ‘chatroom*.

‘Self harm*’, ‘selfharm*’, ‘suicid*’, ‘distress*’, ‘self injur*’, ‘emotion*’, ‘problem*’, ‘suicidal behaviour’, ‘suicidal ideation’, ‘attempted suicide’, ‘self-injurious behaviour’, ‘self mutilation’, ‘automutilation’, ‘psychological stress’

‘Adolescen*’, ‘child*’, ‘teenager*’, ‘young person*’, ‘young adult*’, ‘young people*’

In the first stage of screening reference titles (n = 4,313) were manually screened by one reviewer (KD). Titles that clearly had no relevance to the study were discarded; and all discarded titles were confirmed by a second reviewer (PM). Duplicates were removed and the remaining titles with abstracts (n = 1830) were divided between five reviewers for screening, with all titles screened by KD. Studies were forwarded to the second stage of screening if they met the following criteria:

published paper in journal or book;

research study;

theoretical/non-theoretical paper;

population was young people below age 25 (young adults);

and included use of the internet by individuals who experienced suicidal ideation, self-harm, or were suicidal.

Papers forwarded to the second stage of screening (n = 225) were categorised by two reviewers according to the criteria in Table 1. Any discrepancy between categorisation was resolved through group consensus. Papers in category 1 (n = 16), i.e. that investigated the internet and self-harm or suicide **and** reported empirical data in a sample under the age of 25, were forwarded for detailed analysis of their methodology and content. Quality was assessed according to the Critical Appraisal Skills Programme [18]. This programme assesses in detail the following aspects: population studied (e.g. how representative the population is, whether there are clear inclusion and exclusion criteria, and presence of any biases); data collection (e.g. whether the methods are clearly described, use of validated instrument, inter-observer and intra-observer biases); study design (e.g. appropriate methodology which is clearly stated); and results (e.g. whether the results are clearly outlined, confounding variables are accounted for and conclusions reflect the analysis). A quality rating for each paper of low, medium or high was obtained using these quality standards.

**Table 1:** Criteria used for initial screening.

Code	Decision	Details
**1**	Include for data extraction	Investigates internet and self-harm or suicide
		**AND**
		Reports empirical data in sample under age 25
**2**	Studies of relevance but without empirical data	Investigates internet and self-harm or suicide
		**AND**
		Discusses possible causes, methods of prevention, risk and protective factors, or theoretical models in sample under age 25 but does not include empirical data
**3**	Use as background literature	Pertains to internet and self-harm or suicide
		**AND**
		Provides useful information on the above
**4**	Exclude as irrelevant to the review	Pertains to internet and self-harm or suicide but does not fulfil above criteria
		**OR**
		Does not pertain to internet, self-harm or suicide in adolescence/young adulthood

### Method of Analysis

The papers were grouped into three categories according to the methodology used: qualitative, quantitative or mixed methods. A data extraction sheet, developed by the authors, was used to record specific findings, identify themes, and ascertain potential biases, limitations and weaknesses. The data extraction sheets and quality ratings were double checked and any inconsistencies were clarified by consensus of all the reviewers (all authors). The data extraction sheets were then examined systematically to identify the results. The studies were amalgamated and grouped according to their perceived influence, either negative or positive.

## Results

**Figure 1:**
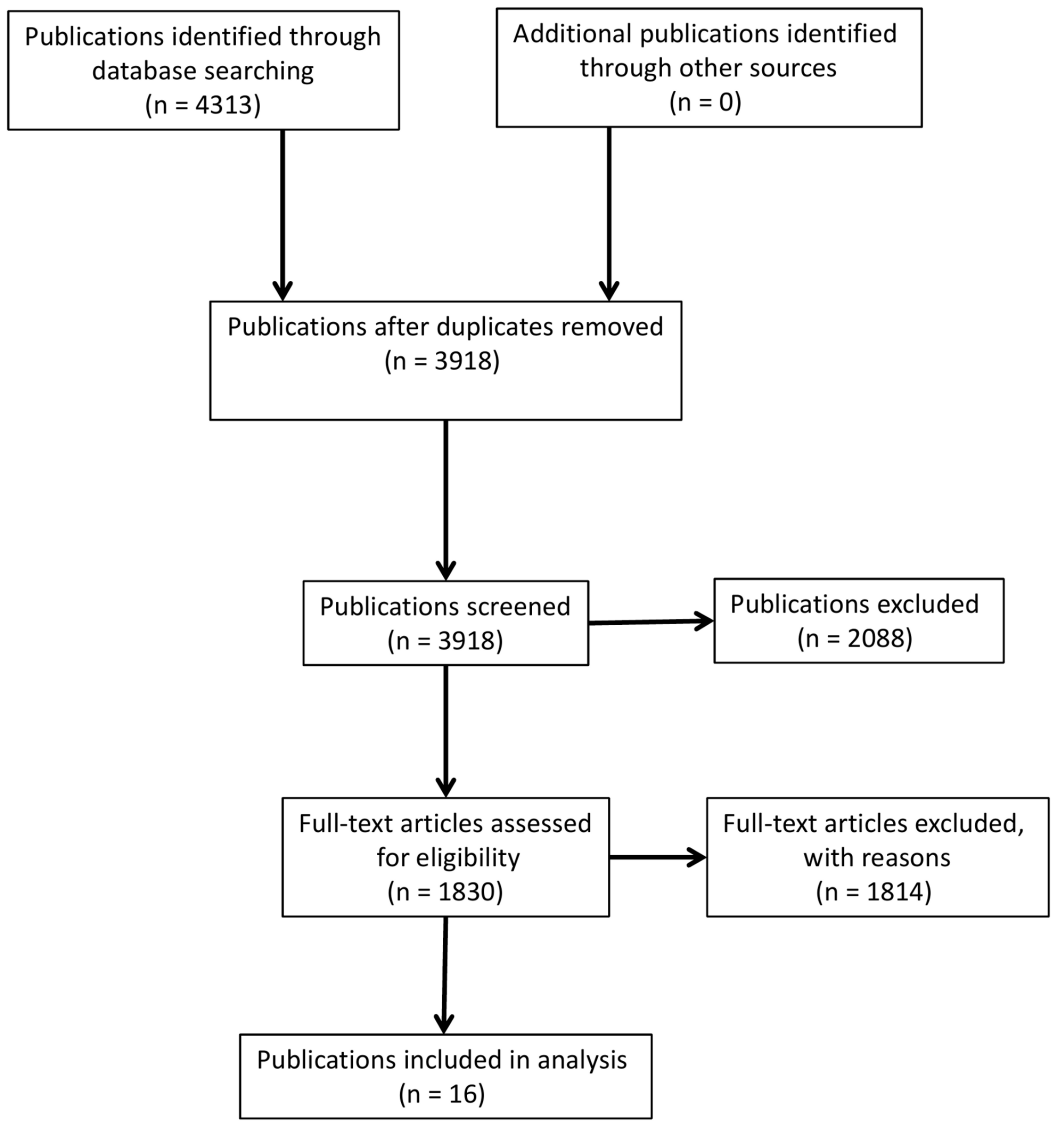
Flow chart of citations from the initial search to the final number of included publications.

A flowchart for results of the search strategy is detailed in Figure 1. Whilst many of the papers that passed the first screen were of some relevance, only 16 papers reported primary empirical data and were forwarded for data extraction. A total of 14 studies were reported in the 16 papers included in the review. The characteristics and content of these papers are summarised in Table S1. Six studies were from the USA, two from the UK/Ireland and one each from Germany, Israel, New Zealand, China, Japan and Korea. The methodology was predominantly quantitative, this approach being used in nine studies, a qualitative approach in two studies, and mixed methods in the remaining three.

### Quality of Included Studies

The quality of the included studies, using rigorous quality assessment and ratings, varied, with eight of the 14 studies being rated as high quality (Table S1), two rated as medium quality and six rated as being of low quality. Almost all the studies relied on subjective data from self-reporting in the form of questionnaires on and off line and semi-structured and structured interviews. Those studies that did not use this form of data collection were observational studies that analysed “posts” left on forums; these had the least potential for bias.

Several studies were limited to active users, such that the results are not necessarily generalisable to passive users [19], [20], [21], [22], [23], [24]. Three of the four qualitative studies report on the same dataset [19], [20], [21] and also did not acknowledge the likely impact of forum moderation by the authors themselves. Criteria for the removal of posts that were deemed ‘triggering’ or ‘challenging’ were also not defined. The authors of one of these studies generalised their aim to explore what young people who self-harm think about online discussion forums, yet the results were specific only to the population that used forums, not the general population of young people who deliberately self-harm as stated (19). Some studies lacked clarity concerning whether the influence of the internet was positive, negative or neutral [20], [21], [25], [26]. Furthermore, the use of validated outcome measures was limited and sometimes the measures selected did not enable the interpretation of internet influence [19].

Despite limitations, it should be noted that often studies were highly innovative and employed novel methodologies to gain access to a vulnerable and hard-to-reach population [24], [27]. For example, in one study in-depth semi-structured interviews were conducted via email to enable a less intrusive mode of response [22]. Furthermore, five of the studies included a study population of over 1000 participants [26], [28], [29], [30], [31].

In reporting the findings we have grouped these into studies providing evidence of positive influences and those showing negative influences, with the specific type of media use described within each category. Positive influences are defined as results indicating perceived alleviation or reduction of psychological distress, including reduced suicidal ideation and actual or attempted self-harm. Negative influences are defined as results which reported or interpreted psychological distress, self-harm or suicidal ideation of any kind. This includes cyber-bullying, which was reported in two of the papers. It should be noted that some findings from the studies cannot easily be categorised as negative or positive. They require further investigation to understand if they may act as moderators or mediators of distress. They may not be either of these, but may help to clarify the influence of the internet on the risk of suicide and self-harm in young people.

Internet use is described in relation to the use of internet forums, or general internet use including information websites, media articles, blogs and static non-interactive media. Any deeper categorisation was prohibited due to lack of reference to specific types of internet media in the results of the studies.

### Positive Influences

Seven studies reported positive influences of internet forums, including four qualitative studies, two mixed methods studies and one quantitative study.

Internet forums are a type of community [22], with membership created by participants through narratives and discussions promoting opportunity for involvement [20]. Primary objectives of forum users are to develop relationships and connect with others [21], and to seek empathy and support rather than advice [22]. Of 3219 posts on an internet forum informal support was the most common type of exchange (28.3%) [24]. Forums have also been used to meet people with similar problems [27] and more generally as a coping mechanism [19], [22], [24]. The total number of posts created and received in the first two months of forum use was associated with lower levels of distress in the third month [23]. Evidence was found for reinforcement of positive behaviours, including congratulations on not cutting, support for efforts not to self-harm, and encouragement to see GPs for help [21]. However, there was no consensus amongst participants that forums prevented a reduction in self-harm or that membership increased the likelihood of self-harm [19]. Forum anonymity was emphasised as beneficial and preferable, and participants reported that more knowledge could be gained from forums than from information sites.

In two studies potentially positive influences of other internet media were found. In one it was suggested that youth reporting self-harm may be using the internet to connect with others and that this may alleviate psychological distress [26]. In the other, evidence was presented that some participants viewed interactive media as a form of support [25].

### Negative Influences

In five studies there was evidence of negative influences of internet forums. In one study self-harm appeared to be discussed in a routine and potentially normalising manner [21]. Safety and empathy were key themes but not the prevention or reduction of self-harm. In another study, forum content regarding the concealment and effects of self-harm accounted for 9.1% (n = 292) of the total posts analysed [24]. There was also an association between sharing self-harm techniques and negative attitudes towards disclosure. Involvement for destructive reasons was endorsed by 14.5% (n = 24) of forum users and 18% (n = 30) stated that finding a suicidal partner had some relevance to them [27]. Discussion forum use was significantly associated with increases in suicidal ideation whereas social networking site use was not [32]. It was also noted that hopelessness was related to increased forum use. In the last of the studies in this category, although lower levels of distress were correlated with forum use, participants overall did not report a decrease in distress and some participants showed signs of worsening distress [23].

Negative influences of other internet media were found in seven studies. General internet use appears to be a source of exposure to both suicide and self-harm, with 59% (n = 429) of participants in one study saying they had learned about suicide from an online source [32]. Of 15 adolescents who had carried out violent acts of self-harm in New Zealand, 80% had been exposed to suicide or self-harm-related material on the internet, and of 34 who self-harmed by cutting 73.5% had been exposed to such material [25]. In another study, suicidal ideation was significantly associated with searching online for information about suicide [33].

Adolescents who self-harm appear to have higher rates of internet use than other adolescents [26], and internet use or online gaming exceeding five hours per day was associated with suicidal ideation and planning [30]. Moderate or severe addiction to the internet was associated with increased risk for self-harm [28], and increased levels of addiction were related to increased depression and suicidal ideation [31].

We found two quantitative studies focused on cyber-bullying and self-harm. In one study it was suggested that cyber-bullying may have a significant influence on self-harm, and that this association may be mediated by negative emotions [34]. In the second study, cyber-bullying also appeared to increase rates of attempted suicide for both victims and perpetrators, with rates increasing by 1.9 and 1.5 times respectively [29]. Forms of cyber-bullying occurred most often via email (18.3%), instant messaging (16.0%), MySpace (14.2%), and chat rooms (10.0%).

## Discussion

We have conducted what is to our knowledge the first systematic review of empirical evidence on potential influences of the internet on adolescents at risk of self-harm or suicide. This is clearly an area of research that is in its infancy, with just 14 studies meeting the criteria for this review. Of the included studies, eleven were conducted in Western countries, the remaining three being from Asia. The predominant research methodology was quantitative. The methodology in just over half of the studies was of high quality, with nearly half of the studies having low or medium quality methodology. Greater heed was paid to studies of higher quality.

The results indicate both positive (9 studies) and negative influences (12 studies) from the internet, with some studies reporting both negative and positive influences. Specifically, some users of online forums perceive them as supportive communities that provide a means of coping [19], [20], [21], [22], [24], [27]. Equally, use of online forums can be associated with feelings of hopelessness and increases in suicidal ideation [32], and it is unclear whether forums alleviate distress [23], [27]. Interactions on online forums may reduce the threshold for self-harming by normalisation of the behaviour [21]. More general internet use seems to be a source of exposure to suicide and self-harm material, and higher usage rates appear to be associated with increased depression, suicidal ideation and self-harm. In assessing the results it is important to consider the focus of the study, the quality of the methodology and the methods used, all of which may influence the results. Of the nine studies reporting positive influence seven of these related to internet forums rather than general internet use. It may be easier to access young people who are using an internet forum, indeed three of the papers refer to one dataset relating to an internet forum which was created by the researchers. Only two of the studies reporting positive influence used purely quantitative methodology: the others used qualitative (five) or mixed methods (two). This was in contrast to the studies reporting negative findings, where eight out of twelve employed quantitative methods. Interestingly, the qualitative studies reporting negative influences also reported positive influences, perhaps highlighting a greater range of perspectives from the qualitative studies. Of note, the quantitative studies mainly appeared to be of high quality, an important factor to take into account when assessing the mostly negative influence reported in these studies. Muehlenkamp [35] similarly found that the specific methodology used in studies influenced prevalence figures for self-harm behaviour reported in adolescent populations.

### Limitations

Only English language publications were included. We did not examine the grey literature or contact experts in the field to find additional studies, thus there may be some publication bias.

Much of the research was focused on the use and influence of internet forums. It is important to note that internet forum users are a self-selecting population, and the extent to which the internet attracts adolescents with elevated distress levels and susceptibility to its negative effects is unknown. Furthermore, it is difficult to investigate the effects of internet forums on passive users, who may comprise a notably different group of adolescents. Validated outcome measures were rarely used, which limits the ability to interpret significant results and draw clear conclusions [19].

It is important to highlight that while in several studies associations between specific types of internet use and self-harm, suicidal ideation and hopelessness were identified, it was not possible to disentangle cause from effect. For example, while more extensive internet use was found to be associated with self-harm, this does not mean that internet use caused the self-harm. On the other hand, it would be difficult and unethical to manipulate internet use and examine its effects on self-harm.

### Implications for Future Research

More rigorous research is needed to clarify the positive and negative influences of the internet on young people at risk of self-harm and suicide focusing on the mediating and moderating factors, in order to harness the benefits and minimise the negative impact.

Research concerning online forum users included primarily female samples. The potential impact of gender should be investigated in future research, to determine the extent to which effects may vary between the genders.

The association between internet use and violent methods of self-harm [25], and the impact of exposure to self-harm and suicide material online [32], should be further investigated. It is unclear which form of internet media may be most influential. For example, more violent or graphic imagery might have greater effects on psychological processes but currently this is unknown. Future research could have important implications for informing policy regarding internet censorship to reduce exposure to harmful materials.

There are no known online interventions to date that specifically target young people at risk of self-harm or suicide. Given the suggestion that adolescents who self-harm are frequent users of online forums [26], future research should investigate possible interventions that could utilise this online medium to minimise the risk of self-harm.

## Conclusions

The purpose of this review was to analyse the empirical evidence to date on the possible influence of the internet on the risk of suicide or self-harm in young people. The dynamic and rapidly evolving nature of the internet means that research will always lag behind actual developments. The internet appears to have potential to exert both a positive and a negative influence on vulnerable young people. The internet may normalise self-harm, provide access to suicide content and violent imagery, and create a communication channel that can be used to bully or harass others. Conversely, the internet is also used as a support network and a coping mechanism, and can connect people who are socially isolated.

Detailed enquiry about internet use should be included in clinical assessment of young people at risk of self-harm or suicide. This is particularly important given evidence that exposure to others who are self-harming is a major risk factor for self-harm [36], [37], [38], the fact that such exposure may occur through the internet, and that exposure to self-harm and suicide on the internet may be associated with potentially more dangerous methods of self-harm [25].

This review highlights the fact that the internet is dynamic and driven by its users; its accessibility and increasing use suggest that it could be an effective mode of intervention delivery. For example, interactive forums run by healthcare workers may be a possible strategy for informing and supporting young people to minimise the risk for self-harm.

## Supporting Information

Table S1 **Included studies: characteristics, methods, aims, outcomes and quality.**
(DOC)

Checklist S1 **PRISMA Checklist.**
(DOC)
