# Supplementary material for: The Power of the Web: A Systematic Review of Studies of the Influence of the Internet on Self-Harm and Suicide in Young People
Source: PLoS One. 2013 Oct 30;8(10):e77555. doi: 10.1371/journal.pone.0077555 (PMC3813687; doi:10.1371/journal.pone.0077555)
Supplement: Table S1 — Included studies: characteristics, methods, aims, outcomes and quality. (DOC) [file pone.0077555.s001.doc]

| **Author**  **Year**  **Country** | **N**  **Age**  **Gender % Female** | **Type of Study** | **Data Collection Method and Response rate** | **Aims, Outcomes, and Quality** |
| --- | --- | --- | --- | --- |
| **Jones****  **2011**  **UK/Ireland** | 77  16-25  96% | Cohort  Qualitative | Online survey 60% | **Aims:** To establish views of self-harm forums. **Outcomes:** Small and large forums were well perceived. Anonymity important and forums considered helpful but need for moderators was expressed. **Study Quality:** Low. |
| **Smithson****  **2011a**  **UK/Ireland** | 77  16-25  96% | Cohort  Qualitative | Extracting themes from forum posts NS | **Aims:** To understand online peer interactions to inform service delivery for youth who self-harm . Attempted to investigate forums as safe sources of support; inappropriate measures and heavy moderation of the site precluded this. **Outcomes:** Support and advice were the driving factors of user participation. **Study Quality:** Low. |
| **Smithson****  **2011b**  **UK/Ireland** | 77  16-25  96% | Cohort  Qualitative | Extracting themes from forum posts NS | **Aims:** To investigate possible support provided by self-harm forums. **Outcomes:** *Ps actively shaped the forum. Discussions focused on narratives and advice on safe methods of DSH. Evidence of positive reinforcement of attempts to control or reduce DSH. **Study Quality:** Low. |
| **Baker**  **2008**  **UK** | 10  18-33  90% | Cohort  Qualitative | In depth semi-structured Email interviews NS | **Aims:** To explore the accounts of young people who self-harm and use forums. **Outcomes:** Forums were used positively for support and communication. Some reduction in incidence of DSH based on subjective and retrospective reports. **Study Quality:** Low. |
| **Barak**  **2006**  **Israel** | 20  NS  75% | Retrospective cohort  *MM | Analysis of posts NS | **Aims:** To assess whether the degree of forum involvement affected distress levels. **Outcomes:** Overall Ps levels of distress did not improve over 3 months. Level of involvement was significantly associated with lower levels of distress. **Study Quality:** Low. |
| **Whitlock**  **2006**  **USA** | 10 MB*  13-22  >74% | Prevalence/ Correlational  MM | Analysis of posts  NS | **Aims:** To investigate the prevalence and nature of self-injury forums, to explore content of discussions, the role of forums in information delivery, and their influence on help-seeking. **Outcomes:** 406 ‘boards’ were found. Informal support was the most common type of exchange. Concealment of practice, perceived addictiveness and formal help-seeking were also discussed. **Study Quality:** High. |
| **Collings**  **2011**  **NZ** | 71  13-25  79% | Cross sectional  MM | Structured interview  NS | **Aims:** To describe the influences of media on suicidal behaviours, from the perspectives of young people. **Outcomes:** Some interactive media was considered supportive by Ps. Also found that 80% (n=12) of those who used violent methods of self-harm had been exposed to suicide content via the internet before the incident. **Study Quality:** High. |
| **Hay**  **2010**  **USA** | 426  10-21  50% | Cross sectional  Quantitative | School survey  93% | **Aims:** To investigate the etiology of self-harm and generate hypotheses based on Agnew’s general strain theory. Predominant focus was on traditional bullying. **Outcomes:** Cyber-bullying was positively correlated with self-harm and suicidal ideation. Not all confounding factors were considered in the results. **Study Quality:** Medium. |
| **Hinduja**  **2010**  **USA** | 1963  10-16  50% | Cross sectional  Quantitative | School survey  96% | **Aims:** To determine if peer aggression is correlated with suicidal thoughts and attempts. Focused on cyber and traditional bullying. **Outcomes:** High correlations between suicidal ideation and bullying both victimisation and offending. **Study Quality:** High. |
| **Kim**  **2006**  **Korea** | 1573  15-16  64.6% | Cross sectional  Quantitative | School survey  NS | **Aims:** To elucidate the relationship between internet addiction, depression, and suicidal ideation. **Outcomes:** Internet addiction scores were positively correlated with suicidal ideation. The specific form of internet media that the Ps were referring to was not measured, only internet use in general. **Study Quality:** High. |
| **Katsumata**  **2008**  **Japan** | 590  NS  49% | Cross sectional  Quantitative | School survey  89.1% | **Aims:** To investigate the association between the experience of using electronic media and suicidal ideation in Japanese adolescents. **Outcomes:** Suicidal ideation was significantly associated with anxiety or emotional pain related to the use of electronic media, adolescents’ suspicions regarding friends and adults, and searching the net for information about suicide. Confounding variables, such as known risk factors for suicide, were not measured. Specific results of internet searches were not reported, nor were the specific media types of internet. **Study Quality:** Low. |
| **Lam**  **2009**  **China** | 1639  13-18  55% | Cross sectional  Quantitative | Survey  98.7% | **Aims:** To examine the association between internet addiction in adolescents and self-harm . **Outcomes:** Ps who were moderately or severely addicted to the internet had a higher likelihood of incidences of self-harm . The study has little relevance to this review as it does not elaborate on the specific online media that may be influencing behaviour. It reinforces general evidence of a correlation between internet use and self-harm . **Study Quality:** High. |
| **Dunlop**  **2011**  **USA** | 719  14-24  51% | Cohort  Quantitative | Telephone/online survey  58% | **Aims:** To determine whether internet sites expose young people to suicide stories that might increase suicidal ideation. **Outcomes:** Youth who use forums were more likely to report suicidal ideation, and youth experiencing hopelessness or who had heavy internet use were more likely to use forums. **Study Quality:** High. |
| **Eichenberg**  **2008**  **Germany** | 164  NS  50% | Cross sectional  Quantitative | Online survey  NS | **Aims:** To assess the assumption that suicide message boards are harmful. **Outcomes:** 48% of users were online for constructive reasons. Destructive use was possibly under-reported. **Study Quality:** High. |
| **Messias**  **2011**  **USA** | 18573  14-18  49% | Cross sectional  Quantitative | School survey  71% | **Aims:** To test the relationship between time spent on video gaming/internet and reported suicidal ideation and depressive symptoms. **Outcomes:** >5 hours use per day was associated with sadness and suicidal ideation. However there Is no distinction between video gaming and internet use so the study has little relevance to this review. **Study Quality:** Medium. |
| **Mitchell**  **2007**  **USA** | 37: 1500  10-17  81% | Cross sectional  Quantitative | Structured interview  45% | **Aims:** To explore internet use and interpersonal interactions of youth reporting self-harm . **Outcomes:** Youth engaging in self-harm were significantly more likely also to have a close relationship with someone met online, and to use chat rooms and instant messaging. **Study Quality:** High |

**** Three studies taken from one dataset *MB = Message Board *NS = Not Stated *Ps = Participants *MM = Mixed methods study**
